# Eating eggplants as a cucurbit feeder: Dietary shifts affect the gut microbiome of the melon fly *Zeugodacus cucurbitae* (Diptera, Tephritidae)

**DOI:** 10.1002/mbo3.1307

**Published:** 2022-08-16

**Authors:** Wouter Hendrycks, Hélène Delatte, Laura Moquet, Kostas Bourtzis, Nele Mullens, Marc De Meyer, Thierry Backeljau, Massimiliano Virgilio

**Affiliations:** ^1^ Department of Biology Royal Museum for Central Africa (RMCA) Tervuren Belgium; ^2^ Evolutionary Ecology Group, Department of Biology University of Antwerp Wilrijk Belgium; ^3^ UMR PVBMT CIRAD Saint‐Pierre La Réunion France; ^4^ Insect Pest Control Laboratory Joint FAO/IAEA Centre of Nuclear Techniques in Food and Agriculture Vienna Austria; ^5^ OD Taxonomy and Phylogeny Royal Belgian Institute of Natural Sciences (RBINS) Brussels Belgium

**Keywords:** gut microbiota, host shift, plant–insect interactions, Tephritidae, wild populations

## Abstract

While contemporary changes in feeding preferences have been documented in phytophagous insects, the mechanisms behind these processes remain to be fully clarified. In this context, the insect gut microbiome plays a central role in adaptation to novel host plants. The cucurbit frugivorous fruit fly *Zeugodacus cucurbitae* (Diptera, Tephritidae) has occasionally been reported on “unconventional” host plants from different families, including Solanaceae. In this study, we focus on wild parental (F_0_) adults and semiwild first filial (F_1_) larvae of *Z. cucurbitae* from multiple sites in La Réunion and explore how the gut microbiome composition changes when this fly is feeding on a noncucurbit host (*Solanum melongena*). Our analyses show nonobvious gut microbiome responses following the F_0_–F_1_ host shift and the importance of not just diet but also local effects, which heavily affected the diversity and composition of microbiomes. We identified the main bacterial genera responsible for differences between treatments. These data further stress the importance of a careful approach when drawing general conclusions based on laboratory populations or inadequately replicated field samples.

## Graphical abstract

We investigated how the gut microbiome composition changes in a cucurbit‐feeding fly *Zeugodacus cucurbitae* when it feeds on atypical solanaceous hosts instead of typical cucurbit hosts. Wild parental (F_0_) adults and semiwild first filial (F_1_) larvae of *Z. cucurbitae* collected from two locations in Réunion Island showed heterogeneous microbiome responses across host plants. This study shows how local processes and host plants can strongly affect the composition of the insect microbiome and the importance of using adequately sampled populations.

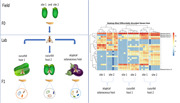

## 1 INTRODUCTION

Insects are the most diverse group of Eukaryotes (Forister et al., 2015) with a vast variety of species being phytophagous and functionally classified in polyphagous, oligophagous, and monophagous when feeding on plants from multiple families, a single family or a single species, respectively (A. R. Clarke, 2017). Contemporary host plant feeding preferences are generally well defined but shifts in host plant preferences have been reported in a variety of insects, such as lepidopterans, beetles, and grasshoppers (Adams et al., 2013; Brown et al., 2014; Rosenberger et al., 2018; Singer & Parmesan, 2021; Sword et al., 2005). The shift toward novel hosts results in ecological niche expansion and subsequent adaptation to the new host can promote genetic divergence between populations, possibly with the evolution of host races and eventually of new species (Feder et al., 1988; Tilmon, 2008). Host races have been reported in a variety of insects, such as beetles and grasshoppers (Lefort et al., 2014; Sword et al., 2005), and a classical textbook example of the evolution of host races is *Rhagoletis pomonella* (Diptera, Tephritidae), where host‐shifts from hawthorn to apples have led to the evolution of genetically divergent populations with different feeding preferences (Feder et al., 1988). Host plant shift or expansion may also favor geographic range expansion (and vice versa) due to the possibility of occupying novel ecological niches and a wider geographic distribution (Hood et al., 2020; Lefort et al., 2014; Rosenberger et al., 2018; Singer & Parmesan, 2021). These processes are of major significance in agronomy and conservation biology as they can promote the emergence of new invasive species and agricultural pests (Brown et al., 2014; Lefort et al., 2014; Lu et al., 2011). The host shift of *R. pomonella* is considered the main cause of the expansion of this species in the Northwest Pacific (Hood et al., 2020).

While changes in insect feeding preferences have been documented in phytophagous insects, the mechanisms behind these processes remain to be clarified. In this context, the insect gut microbiome plays a central role in adaptation to novel host plants as it is of crucial importance for the complex interactions with insect metabolic pathways, which ultimately affect insect fitness (Hammer & Bowers, 2015; Zilber‐Rosenberg & Rosenberg, 2008). The gut microbiome of phytophagous insects can help to (1) break down the complex polysaccharides of the host plant cell wall, and (2) supplement nitrogen, vitamins, and sterols to nutritionally poor diets (Ben‐Yosef et al., 2010, 2014; Douglas, 2009), and (3) detoxify host plant allelochemicals (Hammer & Bowers, 2015) and insecticides (Ishigami et al., 2021; Kikuchi et al., 2012). For example, the symbiotic bacterium “*Candidatus* Erwinia dacicola” is essential for the metabolism of larvae of the olive fly *Bactrocera oleae* (Rossi, 1790) (Diptera, Tephritidae) as it allows them to feed on unripe olives rich in allelochemicals (Ben‐Yosef et al., 2015; Pavlidi et al., 2017). The geographic range expansion of the kudzu bug (*Megacopta cribaria*, Hemiptera: Plataspidae) in the United States has been related to genomic mutations in its symbiont *Ishikawaella*, which allowed the insect to attack soybean as a novel host plant (Brown et al., 2014). Likewise, the invasive spread of some bark beetle species (Coleoptera: Scolytinae) has been associated with compositional changes in their microbial and fungal symbionts (Adams et al., 2013; Lu et al., 2011).

Tephritid fruit flies are a diverse group of flies with herbivorous larvae, with several species being notorious agricultural pests (Norrbom et al., 1999). Recent studies have started to investigate the importance of their gut microbiomes. However, as most of these studies target laboratory colonies with depauperate microbiomes, their results might not be fully representative of what happens in the field (Augustinos et al., 2019; Morrow et al., 2015). For example, laboratory lines of the olive fly, *Bactrocera oleae* often lack “*Candidatus* Erwinia dacicola” despite this symbiont is crucial for larval survival and development on the olive host (Augustinos et al., 2019; Ben‐Yosef et al., 2015; Kounatidis et al., 2009). Conversely, *Acetobacter*, *Morganella*, and *Paenibacillus* can be found in laboratory lines of *B. oleae*, while these bacteria are not a relevant component of the gut microbiome of wild populations (Kounatidis et al., 2009). In this respect, De Cock et al. (2020) reported that gut microbiomes of wild tephritid agricultural pests are highly heterogeneous and stressed the importance of local effects in shaping gut microbiome diversity.

In this study, we focus on wild parental (F_0_) adults and semiwild (F_1_) larvae of the frugivorous fruit fly *Zeugodacus cucurbitae* (Coquillett, 1899) (Diptera, Tephritidae). This widespread agricultural pest is commonly found in South and East Asia, Africa, and Hawaii (De Meyer et al., 2015; Virgilio et al., 2010). It recently expanded from East to West Africa and the islands in the Indian Ocean including La Réunion (De Meyer et al., 2015; Delatte et al., 2019). Larvae of this oligophagous species typically feed on Cucurbitaceae, but occasionally they also attack a variety of “unconventional” host plants from different families, including Solanaceae (De Meyer et al., 2015; Dhillon et al., 2005; Hafsi et al., 2016; Moquet et al., 2021). This, together with observed geographic range expansion in recent decades (De Meyer et al., 2015), raises concerns about the invasion risk of this species, which might be polyphagous rather than oligophagous.

The objective of this study is to explore how the gut microbiome composition of the cucurbit‐feeder *Z. cucurbitae* changes when it feeds on a noncucurbit host plant and how gut microbiome assemblages could facilitate the use of novel host plants in this phytophagous agricultural pest.

## 2 MATERIALS AND METHODS

### 2.1 Experimental design, wet and dry laboratory procedures

In August 2019, infested, wild ivy gourd fruits (*Coccinia grandis* L. Voigt) were collected in La Réunion at two sites: Bassin Plat (−21.321457, 55.485044, “BP”) and Manapany (−21.374674, 55.598343, “M”). They were brought to the laboratories at CIRAD (Centre de coopération internationale en recherche agronomique pour le développement) in Saint‐Pierre. Collected fruits were placed in plastic boxes that were covered with fine‐mesh clothes and contained sand as a substrate for pupation. The fruits were kept in a climatic chamber (25 ± 1°C; 80 ± 10% HR; 12:12 light:dark photoperiod, with artificial light) until pupation. Boxes were regularly inspected for the presence of pupae. Pupae were transferred to 30 × 30 × 30 cm cages in the same climatic chamber. A few days later, adults emerged from the pupae. Adults were fed ad libitum with a diet of sugar and hydrolyzed yeast and had access to a wet sponge as a water source. We waited 3–4 weeks until females were mature and mated before the experiment was started. For each of the two sites, 12 adult gravid F_0_ females were randomly assigned to three experimental cages (four females per cage), each containing a single fruit of *C. grandis* L. (Cucurbitaceae, “Co”), or of *Cucumis sativus* L. (Cucurbitaceae host, “Cu”), or *Solanum melongena* L. (Solanaceae host, “So”). The females were let to oviposit and the resulting six groups of third instar F_1_ larvae (one group for each combination of site and host), were subsampled and subjected to gut microbiome profiling. For each fruit, we considered five replicated microbiome profiles. Each profile was represented by pooled DNA extracts from five individual larvae (following preliminary standardization of individual DNA concentrations). Due to the relatively large dimensions of the target fruits and the relatively limited proportion of plant tissue affected by larval activity, we assume that the extent of horizontal bacterial transmission across larvae within fruits was relatively limited and did not substantially bias the microbial patterns observed. To evaluate whether microbiome differences between larvae reflect differences between adults, we also profiled the microbiomes of four F_0_ adult females from each parental group. All specimens were preserved in 100% EtOH at −80°C before microbiome profiling. As De Cock et al. (2019) did not observe significant differences between whole body and gut microbiome profiles, we profiled whole body DNA extracts from larvae and adults. Laboratory procedures followed De Cock et al. (2020) unless indicated otherwise. Larval microbiome profiles were obtained by pooling DNA extracts from five individual larvae, while adult F_0_ females were individually profiled. Metagenomic library preparation and sequencing were outsourced to Macrogen (https://www.macrogen.com). For library preparation, the Nextera XT kit was used (target: V3–V4 regions of 16S rRNA, insert size: ca. 464 bp, primers: 341 F and 805 R (Takahashi et al., 2014). Libraries were sequenced on an Illumina MiSeq. 150 PE platform (300 bp paired‐end sequencing). A negative and positive control (ZymoBIOMICS Microbial Community Standard D6300) were included to check for artifacts in library preparation and sequencing. Results for the mock community can be found in Table A1.

After verifying the quality of reads with FastQC (Andrews, 2014), we used the DADA2 pipeline (Callahan et al., 2016) to remove primers and truncate reads (to a final length of 260 bp for forward reads and 240 bp for reverse reads resulting in ca. 36 bp overlap) and to fit the parametric error model for the identification and filtering of sequencing errors (2 × 10^6^ reads used for model fitting). Filtering was based on maxEE = 1 in DADA2 (the complete DADA2 pipeline is available on GitHub and in Zenodo at https://doi.org/10.5281/zenodo.6810766). For error estimation, the first two million sequences were used in the error model construction. Before pairing forward and reverse reads and filtering out chimeras, unique amplicon sequence variants (ASVs; 100% unique sequence identity) were extracted using the Bayesian classifier method of DADA2. The Silva v.132 database was used for the taxonomic classification of ASVs (percentage of identity = 97% similarity, *p*‐min‐consensus = 0.51). Chloroplast and mitochondrial sequences were removed using the R package decontam (Davis et al., 2018).

Core (stable associates) bacterial genera and ASVs were identified using the abundance–ubiquity method with a 50% minimal ubiquity threshold. This statistic evaluates whether a bacterial taxon is not more abundant than expected for its ubiquity. Significant deviations from expectation indicate that a bacterial taxon is not a stable core member (Hester et al., 2016). Statistical analyses were performed in R unless stated otherwise. Permutational analysis of variance (PERMANOVA) was conducted with PRIMER v7 (K. R. Clarke & Gorley, 2015) using 9999 unrestricted permutations of raw data).

### 2.2 Microbiome diversity and predictive functional profiling

**Figure A1:**
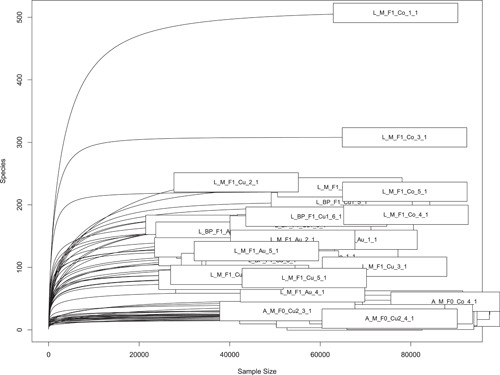
Rarefaction plot showing relationships between sampling depth and the number of bacterial amplicon sequence variants detected.

Three α diversity metrics were calculated: the Abundance coverage estimator (ACE) to assess ASV richness, the Inverse Simpson index (ISI) to assess ASV evenness, and Faith's phylogenetic diversity (FPD) to investigate phylogenetic richness. To evaluate differences in microbial α diversity between larvae raised on different host plants and/or between sites, we used two‐way ANOVAs (Underwood, 1997) which for F_1_ larvae included Site (BP and M) as a random factor and Host Plant (*C. grandis*, *C. sativus*, and *S. melongena*) as a fixed orthogonal factor and for F_0_ adults included Site (BP and M) as a random factor and Parental Group (groups 1, 2, and 3) as a random factor nested in Site. ANOVA was implemented using the GAD package (Sandrini‐Neto & Camargo, 2015). Count data from which diversity metrics were calculated were not normalized as all rarefaction plots reached a plateau (Figure A1). To ensure homoscedasticity, a log transformation was applied to the ISI and a fourth root transformation was applied to the ACE and FPD. Cochran's *C* tests were used to test for homogeneity of variances with the GAD package (Fox, 2006). Pairwise comparisons were done by using an *F* test with Holm correction for multiple comparisons implemented in the phia package (De Rosario‐Martinez, 2013).

Before calculating β diversities, we first removed all ASVs that occurred in only one sample (Chakrabarti et al., 2016; L. J. Clarke et al., 2019) and we normalized counts by transforming them into proportions to represent community structure (McKnight et al., 2019; McMurdie & Holmes, 2014).

Generalized UniFrac distances using the d5 matrix and unweighted UniFrac distances were calculated as β diversity metrics (Chen et al., 2012). As UniFrac distances take into account the phylogenetic relationships, we constructed a midpoint‐rooted maximum likelihood tree of the bacterial relationships using a general time‐reversible substitution model in the program Fasttree (Price et al., 2009). Bacterial 16S sequences were aligned with the DECIPHER algorithm (Wright, 2015).

Differences in microbiome β diversity between larvae raised on different host plants and/or between sites were tested using a two‐way PERMANOVA (Anderson, 2017) with Site (BP and M) as a random factor and host plant (*C. grandis*, *C. sativus*, and *S. melongena*) as a fixed orthogonal factor. The false discovery rate (FDR) correction (Benjamini & Hochberg, 1995) with experiment‐wise *p* < 0.05 was used to correct for multiple testing. PERMANOVA was also used to estimate the components of variance explained by each factor. Differences between larvae raised on different host plants were visualized with a principal coordinate analysis (PCoA) and 95% confidence ellipses were drawn using the ggplot2 package (Wickham & Chang, 2016).

To test for a differential abundance of microbial genera (i.e., genera with relatively more sequences assigned to them) among larvae raised on different host plants and from different sites, we used ALDEx2 (Fernandes et al., 2013). ASVs that could not be classified were assigned to distinct, unidentified genera. Genera that showed differential abundance between two treatments with an effect size difference between 1 and −1 were filtered out to reduce the false positive rate (Gloor, 2015). Significance was assessed by both the Welch *t* test and the Wilcoxon rank‐sum test followed by FDR correction with experiment‐wise *p* < 0.05 as FDR is better suited for exploratory analyses (Lee & Lee, 2018). We selected 23 genera that showed the greatest differences in ALDEx2 to visualize patterns in heatmaps using the pheatmap package (Kolde, 2015). Clustering of sites and diet sources was done using Euclidean distances. For eight of these genera, we constructed boxplots with ggplot and arranged them in a single figure using the ggarrange function in the ggpubr package (Kassambra & Kassambra, 2020).

## 3 RESULTS

The MiSeq Illumina run yielded more than 9.1 × 10^6^ reads (mean per sample = 95,128.91; SD = 15,800.33). After filtering, demultiplexing, merging, and chimera removal, about 3.6 × 10^6^ reads and 2030 unique ASVs were identified. The latter was then assigned to 404 identified genera.

### 3.1 Microbiome composition of adult *Z. cucurbitae*

**Figure A2:**
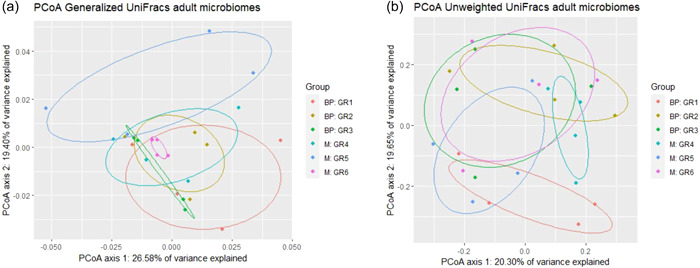
Principal coordinates analysis (PCoA) of microbial communities of wild parental (F_0_) adults of *Zeugodacus cucurbitae* using either (a) Generalized UniFrac distances or (b) Unweighted UniFrac distances. BP_Gr1 = F_0_ adults emerging from *Coccinia grandis* [*C. grandis*] and ovipositing on *Solanum melongena* from Bassin Plat (BP), BP_Gr2 = on *C. grandis* from BP, BP_Gr3 = on *Cucumis sativus* from BP, M_Gr1 = on *S. melongena* from Manapany (M), NDM_Gr2 = on *C. grandis* from M, M_Gr3 = on *C. sativus* from M. The 95% confidence ellipses are shown for each group.

*Enterobacter*, *Klebsiella*, and *Citrobacter* were identified as core genera sensu Hester et al. (2016). In general, microbiomes of adult flies had a mean ASV richness of 26.12 (SD = 8.12) and a mean FPD of 5.97 (SD = 1.01) for adult microbiomes. They were also more uneven (more dominated by few abundant taxa) with an average ISI of 4.95 (SD = 2.25). No differences in any diversity metric were found between F_0_ adults from different sites or different parental groups (Table A2). PERMANOVA of parental F_0_ adult fly microbiomes (Tables A3 and A4) revealed significant differences between parental groups when species presence/absence (unweighted UniFrac distances) was considered (with one out of six significant post hoc comparisons). However, when relative abundances were taken into account and more weight was given to highly abundant taxa (generalized UniFrac distances), the F_0_ parental groups were not significantly different from each other. No significant differences between sites were found for both distances (Table A3). The PCoA plots (Figure A2), could not resolve distinct parental clusters for both unweighted and generalized UniFrac distances.

### 3.2 Microbiome composition of larval *Z. cucurbitae*

**Figure A3:**
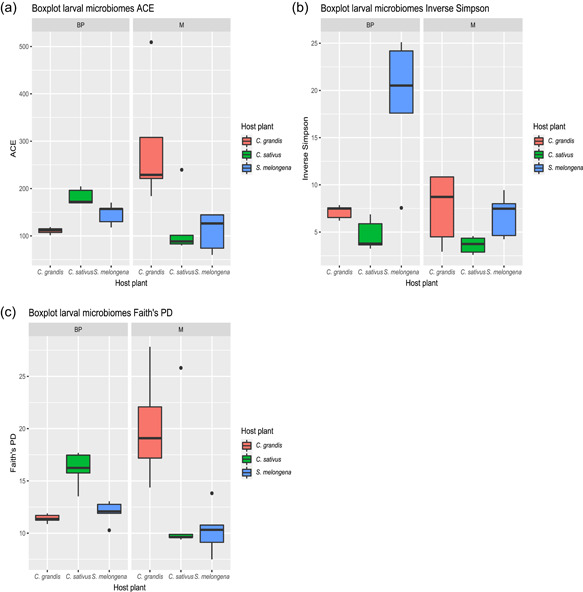
α Diversity metrics: (a) abundance coverage estimator (ACE), (b) inverse Simpson index, and (c) Faith's phylogenetic diversity (PD), calculated from semiwild F_1_ larvae from different host plants (*Coccinia grandis* [*C. grandis*], *Cucumis sativus* [*C. sativus*], and Solanum melongena [*S. melongena*]) and sites (BP: Basin Plat and M: Manapany).

Across all host plants, we identified seven core genera (Hester et al., 2016): *Acinetobacter, Enterobacter, Klebsiella, Paenibacillus, Pseudomonas, Stenotrophomonas*, and *Sphingobacterium*. Larval microbiomes showed a high ASV richness (mean = 145.36, SD = 75.56) but low phylogenetic diversity (mean = 13.51, SD = 4.58). Despite their high ASV richness, larval microbiomes were dominated by very few ASV as the ISI had a mean of 7.97 (SD = 6.21). The log‐transformed ISI did not show consistent patterns across sites or host plants (Figure A3 and Table A5). There was a significant interaction between the host plant and site in the fourth root transformed ACE, the log‐transformed ISI, and the fourth root transformed FPD (Table 1). The α diversity indices did not show consistent patterns across sites and host plants. ASV richness as estimated by ACE and FPD showed significantly higher values for Co in M, but not in BP. Conversely, evenness showed significantly higher values for So in BP, but not in M (Figure A3 and Table A5).

**Table 1:** Analysis of variance on α diversity metrics (abundance coverage estimator, inverse Simpson index, and Faith's phylogenetic diversity) calculated from semiwild F_1_ larvae from different host plants (*Coccinia grandis, Cucumis sativus, Solanum melongena*) and sites (Basin Plat and Manapany).

Abundance coverage estimator	*df*	Mean squares	Pseudo *F*	*p* Value	Effect
Site (Si)	1	0.010	0.132	0.718	Random
Host plant (Ho)	2	0.255	0.219	0.820	Fixed
Si × Ho	2	1.167	14.494	0.000***	
Residual	24	0.080			
				Transformation = Fourth root	
				*C* = 0.365 n.s.	

Inverse Simpson index	*df*	Mean squares	Pseudo *F*	*p* Value	Effect
Site (Si)	1	1.408	9.484	0.005**	Random
Host plant (Ho)	2	2.433	3.896	0.204	Fixed
Si × Ho	2	0.624	4.206	0.027*	
Residual	24	0.148			
				Transformation = Log	
				*C* = 0.385 n.s.	

Faith's phylogenetic diversity	*df*	Mean squares	Pseudo *F*	*p* Value	Factor
Site (Si)	1	0.002	0.216	0.645	Random
Host plant (Ho)	2	0.054	0.444	0.692	Fixed
Si × Ho	2	0.122	9.254	0.001**	
Residual	24	0.013			
				Transformation = Fourth root	
				*C* = 0.602**	

Abbreviations: *C*, Cochran's *C*; n.s., not significant.

* *p* < 0.05;

** *p* < 0.01;

*** *p* < 0.001.

PERMANOVA of the generalized and unweighted UniFrac distances revealed a significant interaction for β diversity between host plant and site (Table 2). However, pairwise comparisons did not provide additional information as all were significant (Table A6). PERMANOVA of the unweighted UniFrac distances showed that the host diet explained 21% of variation, the site explained 18.8% of variation, and 33% of variation was explained by their interaction. For the generalized UniFrac distances, 18% of variation was explained by site, 13.6% of variation by host diet, and 27% by their interaction.

**Table 2:** Permutational analysis of variance on (A) unweighted UniFrac and (B) generalized UniFrac distances calculated from semiwild F_1_larvae from different host plants (*Coccinia grandis*, *Cucumis sativus*, and *Solanum melongena*) and sites (Basin Plat and Manapany).

	*df*	Mean squares	Pseudo *F*	*p* Value	Effect
(A) Unweighted UniFrac distances					
Site (Si)	1	0.573	13.086	0.000***	Random
Host plant (Ho)	2	1.026	1.750	0.031*	Fixed
Si × Ho	2	0.586	13.378	0.000***	
Residual	24	0.043			
Total	29				
(B) Generalized UniFrac distances					
Site (Si)	1	0.5129	23.6740	0.0001***	Random
Host plant (Ho)	2	0.5764	1.4745	0.1157 n.s.	Fixed
Si × Ho	2	0.3909	18.0409	0.0001***	
Residual	24	0.0217			
Total	29				

Abbreviation: n.s., not significant.

* *p* < 0.05;

** *p* < 0.01;

*** *p* < 0.001.

**Figure 1:**
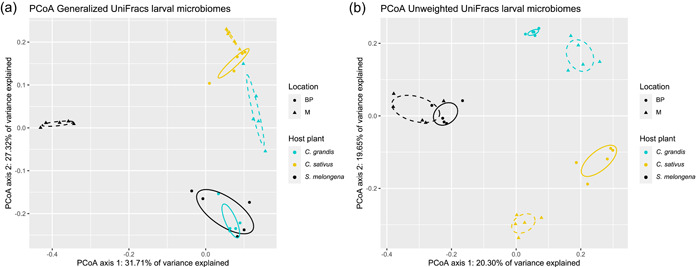
Principal coordinates analysis (PCoA) of microbial communities of semiwild F_1_ larvae of *Zeugodacus cucurbitae* from different host plants (*Coccinia grandis* [*C. grandis*], *Cucumis sativus* [*C. sativus*], *Solanum melongena* [*S. melongena*]) and sites (BP: Basin Plat and M: Manapany) as calculated from (a) generalized UniFrac and (b) unweighted UniFrac distances.

The PCoA on presence/absence data (unweighted UniFrac distances) explains 46.15% of variation and suggests a similarity between larvae feeding on different host plants (Figure 1); however, this pattern is not recovered in the PCoA on weighted abundancies (generalized UniFrac distances, 59.03% of variation).

**Figure 2:**
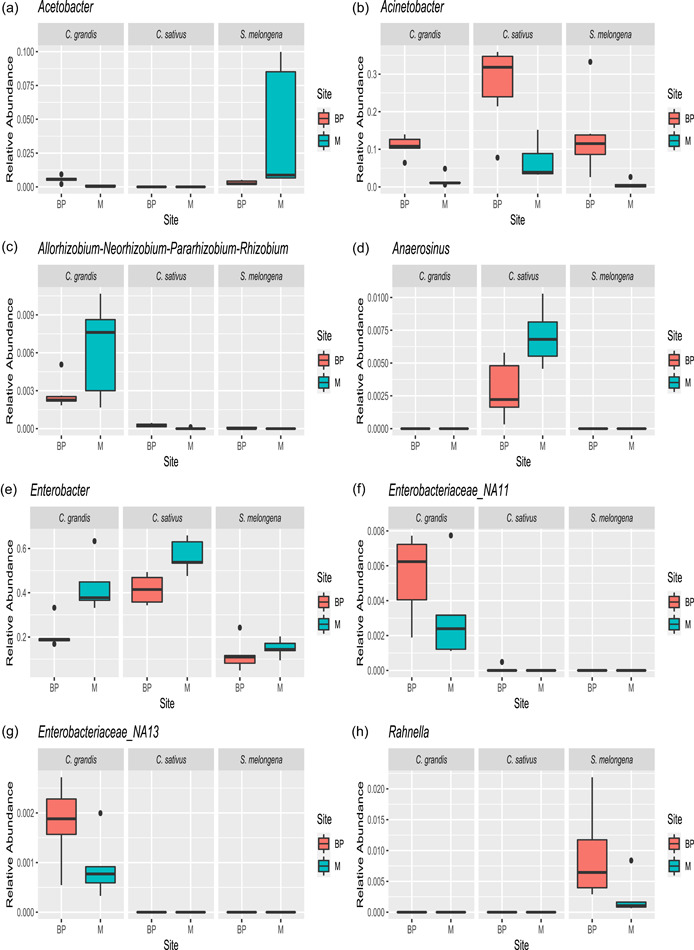
ALDEx2. Relative abundances (%) of bacterial genera in semiwild F_1_ larvae. Results are shown for eight genera (including two unidentified genera of Enterobacteriaceae) with the highest contribution to differences across host plants. BP, Basin Plat; M, Manapany. From left to right and top to bottom: (a) *Acetobacter*, (b) *Acinetobacter*, (c) *Allorhizobium‐Neorhizobium‐Pararhizobium‐Rhizobium*, (d) *Anaerosinus*, (e) *Enterobacter*, (f) *Enterobacteriaceae_NA11*, (g) *Enterobacteriaceae_NA13*, (h) *Rahnella*.

**Figure 3:**
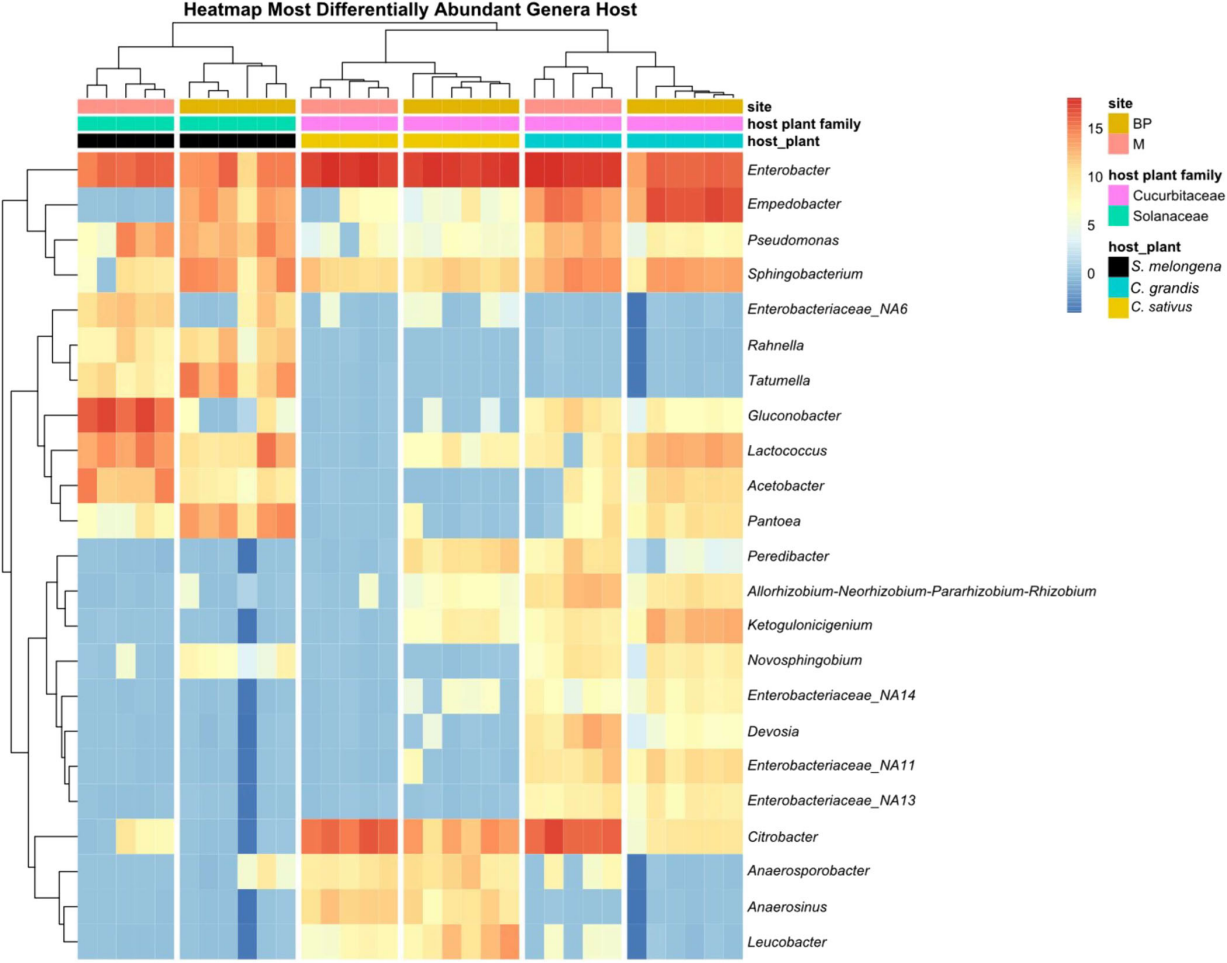
Differential abundances of bacterial genera in semiwild F_1_ larvae across sites (BP: Basin Plat and M: Manapany) and host plants (*Coccinia grandis* [*C. grandis*], *Cucumis sativus* [*C. sativus*], and *Solanum melongena* [*S. melongena*]). Host plant families are indicated in different colors). The red, yellow, and blue colors show the relative abundance of a bacterial genus from high to low. Relative abundances are expressed as centered log‐ratio transformations of the count data.

Several microbial genera showed a significant differential abundance between larvae raised on different host plants (Figures 2 and 3 and Supporting Information table at https://doi.org/10.5281/zenodo.6811204). *Ketogulonicigenium*, *Devosia, Salmonella, Allorhizobium*, and three putative unidentified genera from the family Enterobacteriaceae genera were more abundant on Co than on Cu and So. Most of the genera with higher abundance in So larvae belonged to the Order Enterobacterales, with *Rahnella* being detected in So larvae only. Compared to Cu larvae, larvae on So also showed enrichment in a few genera belonging to the Acetobacteriaceae, the class Bacilli, and the genus *Pseudomonas*. Larvae raised on Cu showed a higher abundance of genera belonging to the bacterial families Clostridia and Negativicutes from the phylum Firmicutes and of the genus *Leucobacter*. An additional three genera of the Clostridia were more abundant in Cu than in Co larvae. In contrast, taxa belonging to Acetobacteraceae and the genus *Pseudomonas* were less abundant in larvae raised on Cu compared to larvae from other host plants.

Between sites, only the genus *Comamonas* showed a pattern of differentiation, as its relative abundance was 8.5× lower in larvae from M than in larvae from BP. Within host plant treatments, more bacterial genera showed differential abundance between M and BP (Supporting Information table at https://doi.org/10.5281/zenodo.6811204).

## 4 DISCUSSION

Although our observations are largely consistent with other tephritid studies, some of our results are not in line with patterns reported in earlier studies (Andongma et al., 2015; Augustinos et al., 2019; Choudhary et al., 2021; De Cock et al., 2020; Morrow et al., 2015), but this is not surprising considering the high variability already described in the microbiome diversity of the closely related genera *Bactrocera* and *Zeugodacus* (De Cock et al., 2020). Part of the variability reported for the microbiome patterns of *Bactrocera*/*Zeugodacus* and more in general for frugivorous tephritids can certainly be related to the different lab rearing and experimental conditions reported in the literature (Asimakis et al., 2019; Augustinos et al., 2019; Ras et al., 2017; Sacchetti et al., 2019). However, discrepancies can also be observed between studies targeting wild or semiwild populations (e.g., Choudhary et al., 2021; De Cock et al., 2020). For example, previous research on wild populations of *Z. cucurbitae* suggested a close association with *Ochrobactrum* (Choudhary et al., 2021; De Cock et al., 2020), while this bacterial genus has an inconsistent presence across our samples.

One of the objectives of this study was to explore changes in the microbiome composition of a cucurbit‐feeder fly feeding on a noncucurbit host (*S. melongena*, Solanaceae) with the expectation that the shift to a noncucurbit diet would produce major and consistent changes in its microbiome. This only partially happened. First, changes in the microbiome composition related to host plant diet mostly concerned less abundant taxa as differences were observed mainly from the analysis of presence/absence data (unweighted UniFrac distances) rather than from the weighted abundances (generalized UniFrac distances). This is consistent with recent studies on termites and wood‐eating cockroaches that also found that dietary shifts mainly resulted in changes in rare microbes while abundant microbial taxa remained generally stable (Benjamino et al., 2018; Pérez‐Cobas et al., 2015). It suggests that low‐abundant species might have a key role in host plant adaptation. Second, our results show that local factors other than diet, have a deep impact on the microbiome diversity of *Z. cucurbitae* and more strongly contribute to the variability of patterns observed. Regardless of the complex interactions between diet and local‐geographical factors, we need to consider that this study mainly focused on F_1_ larvae. So, one explanation for these results might be that several generations are required before reaching stable and consistent microbiome assemblages in flies shifting to a novel “atypical” diet. A recent study in the whitefly *Bemisia tabaci* (Hemiptera, Aleyrodidae), for example, found that an initial host switch from watermelon to the less suitable host pepper did not result in major changes in microbiome composition and structure (Santos‐Garcia et al., 2020). Yet, major microbiome changes did occur in subsequent generations. Moreover, the same study also showed that the first generation following the host shift had a lower survival rate, which increased again in subsequent generations, suggesting that the microbiota was involved in longer‐term adaptation to the new host plant. Similarly, in the diamondback moth (*Plutella xylostella*, Lepidoptera, Plutellidae) a host shift to novel pea hosts resulted in major microbiome changes only in later generations, not in the first generation (Yang et al., 2020).

Another hypothesis for the lack of straightforward relationships between diet and microbiome composition is that microbiome changes mainly involve bacterial taxa that serve important metabolic functions but which are so rare that they remain undetected. Indeed, rare members of microbial communities sometimes perform key functions in these communities (Jousset et al., 2017). *Desulfosporinus*, for example, represents only 0.006% of reads detected in microbial peatland communities and yet it contributes the most to sulfate reduction (Pester et al., 2010). Likewise, the capacity of freshwater microbial communities to degrade pollutants is severely reduced when rare taxa disappear (Delgado‐Baquerizo et al., 2016). So, rare taxa may support a community with a wide range of metabolic functions that might only be important under specific circumstances (Jousset et al., 2017) such as the use of an unconventional host plant species.

We also need to consider that changes in host plant use might not necessarily translate into major compositional changes in microbiome assemblages but rather result in changes in the gene expression patterns of the “holobiont” sensu Margulis and Fester (1991) (i.e., the insect and its microbiota living on the host plant), which is the central unit of symbiogenesis and evolution (Guerrero et al., 2013). Symbiont microbial pectinases complement the insect endogenous cellulases and xylanases in herbivorous beetles (Cassidinae) and the pectinolytic range of symbiotic bacteria of the genus *Stammera* has been associated with the diversity of host plants that can be attacked by these beetles (Salem et al., 2020). Accordingly, the flexibility of insect and microbiome gene expression patterns could provide a complementary/alternative explanation to the complex relationships observed between microbiome assemblages, feeding preferences, and range expansion of *Z. cucurbitae*.

Additionally, differences in microbiome composition and structure between larvae feeding on the noncucurbit host could have also been affected by differences in their parental microbiomes, as significant heterogeneity was detected between the microbiomes of the parental lines and since in tephritids, at least part of the microbiome is vertically transmitted (Behar et al., 2008).

One potential drawback of our study is that we did not investigate the effect of captivity on the fly microbiome, especially with regard to vertical transmission from adults to larvae. Although we used wild populations for our experiment, our setup required the breeding of one generation in captivity. Previous studies have already shown that captive conditions can affect the microbiomes of tephritids (Asimakis et al., 2019; Augustinos et al., 2019; Morrow et al., 2015; Ras et al., 2017; Sacchetti et al., 2019). Moreover, changes in the larval microbiome due to captivity can occur in even relatively short periods. Majumder et al. (2022), for example, already detected changes in larval microbiomes after a single generation in captivity. Interestingly, changes in the adult microbiome took more generations before they started to occur. This suggests that rapid changes in the larval microbiome due to captivity are more due to environmental differences between the captive and natural environment experienced by the larvae (not the adult), such as diet, rather than due to a loss of vertically transmitted symbionts or genetic changes (Majumder et al., 2020). Indeed, prior studies have shown that the larval microbiome undergoes significant changes when larvae are reared on an artificial diet rather than a more natural diet. Likewise, studies on zoo animals have shown that exposing animals to more natural conditions and bacterial sources keeps their microbiome more similar to the microbiomes of their wild relatives (Loudon et al., 2014). Because of this, we do not consider this a major drawback of our study as larvae in our experiment were reared on a natural diet and exposed to a more natural environment, reducing, therefore, the impact of captivity on larval microbiomes and keeping them more representative of the natural conditions.

## 5 CONCLUSIONS

This study describes the effects of parent–offspring host switches in a cucurbit feeder fly and reveals complex microbiome responses in wild populations. As in De Cock et al. (2020), our results stress the importance of local effects on microbiome diversity and composition and the impact that factors such as diet can have on the microbiome. We identified the main bacterial genera responsible for the patterns observed. The high local‐scale variability and its interaction with diet shifts revealed the importance of proper spatial replication in microbiome research targeting wild/semiwild tephritid flies and provide a cautionary tale on general inferences drawn from laboratory populations.

## AUTHOR CONTRIBUTIONS

**Wouter Hendrycks**: formal analysis (lead); funding acquisition (supporting); investigation (supporting); visualization (lead); writing – original draft (lead); writing – review & editing (equal). **Hélène Delatte**: conceptualization (equal); methodology (equal); project administration (equal); resources (equal); supervision (equal); writing – original draft (supporting); writing – review & editing (equal). **Laura Moquet**: investigation (supporting); project administration (supporting); supervision (equal); writing – original draft (supporting); writing – review & editing (equal). **Kostas Bourtzis**: conceptualization (supporting); funding acquisition (equal); writing – original draft (supporting); writing – review & editing (equal). **Nele Mullens**: formal analysis (supporting); investigation (lead); visualization (supporting); writing – original draft (supporting); writing – review & editing (equal). **Marc De Meyer**: conceptualization (equal); funding acquisition (supporting); methodology (equal); resources (equal); supervision (supporting); writing – original draft (supporting); writing – review & editing (equal). **Thierry Backeljau**: conceptualization (supporting); funding acquisition (equal); writing – original draft (supporting); writing – review & editing (equal). **Massimiliano Virgilio**: conceptualization (equal); formal analysis (supporting); funding acquisition (supporting); methodology (equal); resources (equal); supervision (equal); writing – original draft (supporting); writing – review & editing (equal).

## CONFLICT OF INTEREST

None declared.

## ETHICS STATEMENT

None required.

## Data Availability

All raw sequence read data are available from the European Nucleotide Archive under the accession number PRJEB49793: https://www.ebi.ac.uk/ena/browser/view/PRJEB49793. Sample data set, sample metadata, and codes for analysis are available on GitHub: https://github.com/wouterhendrycks/tephritid_microbiome_host_switch_project and in Zenodo: https://doi.org/10.5281/zenodo.6810766. Supporting Information table (Differential abundance analysis of bacterial genera between larval microbiomes from larvae between different host plants and between different sites using ALDEx2) is available in the Zenodo repository at https://doi.org/10.5281/zenodo.6811204).

## APPENDIX A

See Figures A1, A2, A3 and Tables A1, A2, A3, A4, A5, A6

**Table A1:** Mock community standard results showing the taxa detected in the mock community and their relative abundance compared to their expected relative abundance.

Species	Relative abundance	Zymo expected 16S rRNA copy (theoretical)
*Lactobacillus fermentum*	14.1	18.4
*Salmonella enterica*or unclassified	2.8	10.4
*Escherichia coli*or unclassified	30	10.1
*Pseudomonas aeruginosa*	17.5	4.2
*Enterococcus faecalis*	13.4	9.9
*Bacillus subtilis*	9.6	17.4
*Listeria monocytogenes*or unclassified	2.7	14.1
*Staphylococcus aureus*	10	15.5
*Lactobacillales NA*	0.01	
*Enterobacteriaceae NA*	0.005	

**Table A2:** Analysis of variance on α diversity metrics (abundance coverage estimator, inverse Simpson index, and Faith's phylogenetic diversity) calculated from F_0_ adult microbiomes from different host plants (*Coccinia grandis*, *Cucumis sativus*, and *Solanum melongena*) and sites (BP: Basin Plat and M: Manapany).

	*df*	Mean squares	Pseudo *F*	*p* Value	Effect
Abundance coverage estimator					
Site: Si	1	0.671	0.012	0.917 n.s.	Random
F_0_ parental group: Gr(Si)	4	55.126	0.651	0.633 n.s.	Random
Residual	18	84.582			
				Transformation = none	
				*C* = 0.4241 n.s.	
Inverse Simpson index					
Site: Si	1	0.001	0.000	0.993 n.s.	Random
F_0_ parental group: Gr(Si)	2	12.790	2.759	0.059 n.s.	Random
Residual	18	0.028	‐		
				Transformation = none	
				*C* = 0.475 n.s.	
Faith's phylogenetic diversity					
Site: Si	1	1.069	0.631	0.471 n.s.	Random
F_0_ parental group: Gr(Si)	2	1.693	1.687	0.197 n.s.	Random
Residual	18	1.003	‐		
				Transformation = none	
				*C* = 0.172 n.s.	

Abbreviations: *C*, Cochran's C; *df*, degree of freedom; n.s., not significant.

**Table A3:** Permutational analysis of variance on (A) unweighted UniFrac and (B) generalized UniFrac distances calculated for microbiomes from parental F_0_ adults from two sites (BP: Basin Plat and M: Manapany).

(A) Unweighted UniFrac distances	*df*	Mean squares	Pseudo *F*	*p* Value	Effect
Site: Si	1	0.353	1.351	0.223 n.s.	Random
F_0_ parental group: Gr(Si)	4	0.262	1.819	0.005**	Random
Residual	18	0.144			
Total	23				

(B) Generalized UniFrac distances	*df*	Mean squares	Pseudo *F*	*p* Value	Factor
Site: Si	1	0.003	1.752	0.098 n.s.	Random
F_0_ parental group: Gr(Si)	4	0.001	1.154	0.259 n.s.	Random
Residual	18	0.002			
Total	23				

Abbreviations: *df*, degree of freedom; F_0_, wild parental; n.s., not significant.

** *p* < 0.01.

**Table A4:** *A posteriori* comparisons between (unweighted UniFrac distances, see Table A3 (A)) between microbiomes of parental F_0_ adults from two sites (BP: Basin Plat and M: Manapany). Comparisons are shown between groups of adults used to infest three different fruits (Co: *Coccinia grandis*, Cu: *Cucumis sativus*, So: *Solanum melongena*)

Unweighted UniFrac distances post hoc comparison				
Site	Comparison	Average dissimilarity	*t* Value	*p* Value
BP	So–Co	0.632	1.865	0.030*
BP	So–Cu	0.602	1.546	0.056 n.s.
BP	Co–Cu	0.523	1.161	0.206 n.s.
M	So–Co	0.495	0.841	0.743 n.s.
M	So–Cu	0.572	1.193	0.174 n.s.
M	Co–Cu	0.596	1.246	0.111 n.s.

* *p* < 0.05.

**Table A5:** *A posteriori* comparisons between α diversity metrics (abundance coverage estimator, inverse Simpson index, and Faith's phylogenetic diversity) calculated from semiwild; F1 larvae feeding on different host plants (Co: *Coccinia grandis*, Cu: *Cucumis sativus*, and So: *Solanum melongena*) and sites (BP: Basin Plat and M: Manapany).

Site	Host plant	Estimate	*df*	Sum of squares	*F* value	*p* Value
Abundance coverage estimator						
BP	So–Co	0.232	1	0.134	1.669	0.625
BP	So–Cu	−0.205	1	0.105	1.303	0.625
BP	Co–Cu	−0.436	1	0.477	5.923	0.091
M	So–Co	−0.867	1	1.882	23.361	0.000***
M	So–Cu	−0.056	1	0.006	0.082	0.776
M	Co–Cu	0.816	1	1.665	20.664	0.000***
Inverse Simpson index						
BP	So–Co	0.903	1	2.040	13.739	0.005**
BP	So–Cu	1.367	1	4.676	31.491	0.000***
BP	Co–Cu	0.464	1	0.538	3.629	0.137
M	So–Co	−0.035	1	0.003	0.021	0.885
M	So–Cu	0.601	1	0.903	6.083	0.063
M	Co–Cu	0.636	1	1.013	6.826	0.061
Faith's phylogenetic diversity						
BP	So–Co	0.036	1	0.003	0.243	0.625
BP	So–Cu	−0.127	1	0.040	3.069	0.277
BP	Co–Cu	−0.163	1	0.066	5.044	0.136
M	So–Co	−0.321	1	0.258	19.522	0.001**
M	So–Cu	−0.080	1	0.016	1.207	0.565
M	Co–Cu	0.241	1	0.146	11.018	0.014*

Abbreviations: *df*, degree of freedom; n.s., not significant.

* *p* < 0.05;

** *p* < 0.01;

*** *p* < 0.001.

**Table A6:** *A posteriori* comparisons between (A) unweighted UniFrac and (B) generalized UniFrac distances (see Table 2) calculated from semiwild F1 larvae feeding on different host plants (Co: *Coccinia grandis*, Cu: *Cucumis sativus*, and So: *Solanum melongena*) and sites (BP: Basin Plat and M: Manapany).

Population	Host plant	Average dissimilarity	*t* Value	*p* Value
(A) Unweighted UniFrac distances post hoc comparison				
BP	So–Co	0.547	5.733	0.008**
BP	So–Cu	0.636	4.678	0.008**
BP	Co–Cu	0.614	4.494	0.008**
M	So–Co	0.685	3.644	0.007**
M	So–Cu	0.618	4.260	0.008**
M	Co–Cu	0.637	3.961	0.006**
(B) Generalized UniFrac distances post hoc comparison				
BP	So–Co	0.373	3.677	0.008**
BP	So–Cu	0.493	4.005	0.008**
BP	Co–Cu	0.467	4.868	0.008**
M	So–Co	0.582	5.206	0.007**
M	So–Cu	0.532	8.334	0.008**
M	Co–Cu	0.369	3.394	0.006**

Abbreviation: n.s., not significant. ﻿﻿

** *p* < 0.01;
